# Editorial: New Rootstocks for Fruit Crops: Breeding Programs, Current Use, Future Potential, Challenges and Alternative Strategies

**DOI:** 10.3389/fpls.2022.878863

**Published:** 2022-03-23

**Authors:** Sergio Ruffo Roberto, Vittorino Novello, Gennaro Fazio

**Affiliations:** ^1^Department of Agronomy, State University of Londrina, Londrina, Brazil; ^2^Dipartimento di Scienze Agrarie, Forestali ed Alimentari, University of Turin, Turin, Italy; ^3^Plant Genetic Resources Unit, USDA/ARS, Geneva, NY, United States

**Keywords:** rootstocks, fruit, breeding, scion compatibility, breeding programs

## Introduction

Rootstocks are playing an increasingly crucial role in determining orchard efficiency and sustainability in fruit crops. Combining the desirable attributes of two or three different individuals by budding or grafting can produce dramatic effects on growth and productivity (Bowman and McCollum, 2015). The effect of rootstocks on fruit quality in terms of physical traits and internal chemical compositions is well known in several fruit crops. Rootstocks can influence precocity/juvenility, yield, tree size control, biotic and abiotic stress resistance or tolerance, fruit respiratory behavior, crop load and canopy management techniques (Domingues et al., 2021).

There has been major progress made by rootstock breeders in the second half of the last century and the beginning of the present century. The increased breeding activity of rootstock breeders is the reason why a wide range of new rootstocks are available to fruit growers. However, breeding rootstocks for fruit crops is much slower than scion breeding within the same species (Cousins, 2005). This is due to the long testing requirements of rootstocks that reduce the opportunity for comprehensive first stage tests on individual plants compounded by expanding selection criteria for new rootstocks. It is much easier to re-graft a scion than replant an orchard.

The current global agricultural challenges imply the need to generate new technologies and farming systems to cope with the need for sustainability and to face up to climate change. In this context, rootstocks are an essential component for fruit crops in modern agriculture. Currently most rootstocks used are clonally propagated and there are several ongoing efforts to develop these plant materials (Gainza et al., 2015). The aim of this Research Topic was to present the latest results of new rootstocks developed using classic and modern selection techniques and forecast novel applications.

In this context, Rufato et al. examined productive performance of apple cultivars grafted on selected Geneva^®^ series rootstocks under extreme conditions of apple replant disease (ARD) areas in southern Brazil, including “Gala Select” and “Fuji Suprema” apples (*Malus domestica* [Suckow] Borkh.) grafted on “G.202,” “G.210,” “G.213,” and “G.814” rootstocks. It was found that the non-fallow condition does not alter the relative differences in vigor and apple fruit quality among the rootstocks, and the G.210 semi-dwarfing rootstock is an alternative for the immediate conversion of “Gala Select” and “Fuji Suprema” apple orchards under these conditions. Within the same species and topic, Mao et al. explored the potential ARD resistance of “12-2” elite rootstock selection and compared it with “M.9-T337” and “M.26” rootstocks, which are commonly grown in China. Authors found that “12-2” elite rootstock can be used as an important genetic source material for breeding of ARD-resistant apple rootstocks, which will be essential for fundamentally solving the rampant problem of ARD in China.

Moving toward another important tree crop species, i.e., citrus (*Citrus sinensis* L.), Bowman et al. described the USDA's citrus breeding program novel, multi-pronged, strategy termed “SuperSour,” for rootstock breeding and presented its key components and methodologies, along with reference to the historical favorite rootstock sour orange (*Citrus aurantium*), and previous methods employed in citrus rootstock breeding. One of characteristics of this strategy is the rootstock propagation by cuttings and or *in-vitro* methods which avoid the need for nucellar seeds (and the associated juvenility period), increases testing replication and eliminates a 6- to 15-year delay in testing while waiting for new hybrids to fruit. As a result, many of the new “SuperSour” hybrid rootstocks exhibited greatly superior fruit yield, yield efficiency, canopy health, and fruit quality, as compared with the standard rootstocks. Within the same species, Carvalho et al. investigated the effects of fruit maturity on seed quality and seedling performance of “US-802,” “US-897,” and “US-942” citrus rootstocks in Florida, US, including the evaluation of seed germination and nursery performance of the seedlings. Authors found that fruit from all three rootstock varieties can be harvested as early as August without losing any germination potential. In another trial, Cruz et al. evaluated the influence of five rootstocks on the vegetative growth, yield performance, fruit quality, and HLB tolerance of “Emperor” mandarin (*Citrus reticulata* Blanco) under the Southern Brazilian humid subtropical climate. Based on their findings “Cleopatra” mandarin, “Sunki” mandarin, “Swingle” citrumelo, and “Fepagro C-13” citrange were considered more suitable rootstocks for “Emperor” mandarin under such conditions.

Some interesting aspects of grapevine (*Vitis* spp.) rootstocks regarding their tolerance to fungal grapevine trunk diseases (GTDs) were investigated by Ramsing et al. in Spain. Twenty-five rootstocks were screened for xylem characteristics and tolerance to main associated fungi. Authors found differences in all the analyzed xylem traits, and also in DNA concentration for both of the main associated fungi among the tested rootstocks. This finding is an important tool to support future rootstock breeding programs to reduce the detrimental impact of GTDs worldwide.

The rootstock-mediated genetic contributions in recombinant juvenile cacao (*Theobroma cacao* L.) across target traits, specifically cadmium (Cd) uptake, and its correlation with growth and physiological traits, were addressed by Férnandez-Paz et al., in which 320 progenies were used as rootstocks in grafts with two commercial clones (ICS95 and CCN51) commonly grown in Colombia. Authors found that differences in the specific combining ability for Cd uptake were mostly detected in ungrafted rootstocks, or 2 months after grafting with the clonal CCN51 scion. These findings will harness early breeding schemes of cacao rootstock genotypes compatible with commercial clonal scions and adapted to soils enriched with toxic levels of Cd.

Also in Colombia, Cañas et al. assessed how elite “crioulo” “plus trees” of avocado rootstocks (*Persea americana* Mill.) inherit trait variation to their seedling progenies, and whether such family superiority may be transferred after grafting to the clonal scion. The results revealed that that elite “criollo” “plus trees” may serve as promissory donors of seedling rootstocks for avocado cv. Hass due to the inheritance of their outstanding trait values.

Finally, Xiong et al. evaluated in China the graft compatibility of melon cv. Akekekouqi (*Cucumis melo*) grafted onto eight *Cucurbitaceae* species including cucumber, pumpkin, melon, luffa, wax gourd, bottle gourd, bitter gourd, and watermelon. The starch–iodine staining technique was used to predict graft compatibility. Authors found that cucumber and pumpkin are graft compatible with melon, while luffa, wax gourd, bottle gourd, bitter gourd, and watermelon are graft incompatible. Also, it was demonstrated that graft compatibility can be evaluated earlier by the starch–iodine staining technique, supporting breeding programs.

## Author Contributions

SR, VN, and GF: writing—review and editing. All authors contributed to the article and approved the submitted version.

## Conflict of Interest

The authors declare that the research was conducted in the absence of any commercial or financial relationships that could be construed as a potential conflict of interest.

## Publisher's Note

All claims expressed in this article are solely those of the authors and do not necessarily represent those of their affiliated organizations, or those of the publisher, the editors and the reviewers. Any product that may be evaluated in this article, or claim that may be made by its manufacturer, is not guaranteed or endorsed by the publisher.
